# Real-Time Beef Cattle Body Condition Scoring Using EdgeBCS-YOLO: A Lightweight Framework for Edge Deployment

**DOI:** 10.3390/ani16081143

**Published:** 2026-04-09

**Authors:** Zitian Liu, Zhi Weng, Zhiqiang Zheng, Caili Gong, Zhuangzhuang Wang, Jun Wang

**Affiliations:** 1 School of Electronic Information Engineering, Inner Mongolia University, Hohhot 010070, China; 32456156@mail.imu.edu.cn (Z.L.); wzhi@imu.edu.cn (Z.W.); 32356154@mail.imu.edu.cn (Z.W.); 32556177@mail.imu.edu.cn (J.W.); 2 State Key Laboratory of Reproductive Regulation & Breeding of Grassland Livestock, Hohhot 010030, China; 3 Full Mechanization Research Base of Dairy Farming Engineering and Equipment, Ministry of Agriculture and Rural Affairs of the People’s Republic of China, Hohhot 010018, China; 4 Inner Mongolia Key Laboratory of Intelligent Communication and Sensing and Signal Processing, Hohhot 010070, China

**Keywords:** beef cattle, body condition scoring, edge computing, feature enhancement, precision livestock farming

## Abstract

Accurate and scalable body condition scoring (BCS) is important for health monitoring and productivity management in precision livestock farming. However, manual scoring is subjective, labor-intensive, and difficult to standardize, while many automated methods are too computationally demanding for edge deployment in real farm environments. This study proposes EdgeBCS-YOLO, a lightweight object detection framework for real-time beef cattle BCS in unstructured farming scenarios. Built on YOLO11n, it combines Position-Sensitive Feature Fusion (PSFF), a Texture-Aware Star Module (TASM), an Efficient Grouped Detection Head (EGDH), and a Focal and Global Knowledge Distillation (FGD)-based distillation strategy. On a dynamic blurring dataset, EdgeBCS-YOLO achieved 90.8% precision, 82.7% recall, and 88.9% mAP@50. On the NVIDIA Jetson Orin NX Super, it achieved a model size of 3.95 MB, a system FPS of 33.35, and an average inference latency of 13.26 ms. These results suggest that it is a practical and potentially efficient solution for automated BCS on edge devices.

## Simple Summary

Body condition is an important indicator of beef cattle health and management, but manual scoring is slow, subjective, and difficult to use on large farms. This study developed an automatic image-based method for body condition assessment in real farm environments. A lightweight model was designed for efficient use on edge devices and achieved competitive accuracy with real-time performance in practical deployment. These findings suggest that beef cattle body condition can be monitored quickly, consistently, and without direct contact. This approach may help improve feeding management, animal welfare, labor efficiency, and the overall sustainability of beef cattle production.

## 1. Introduction

Body Condition Scoring (BCS) is a systematic method for assessing body energy reserves in beef cattle, reflecting their nutritional, health, and welfare status. Despite its recognized value in matching nutritional supply with animal physiological needs, the application of this technology in commercial beef production remains limited. Clinically, BCS serves as a non-invasive diagnostic aid for identifying individuals with increased susceptibility to disease due to poor body condition, making it vital for herd health management. In production practice, BCS provides a basis for precision feeding and optimized management; by grouping herds based on BCS, the efficient allocation of nutritional resources can be achieved, thereby improving the efficiency of breeding programs. This evaluation system utilizes a 9-point scale with 1-point increments, where a score of 1 represents extreme emaciation and 9 represents excessive obesity [1]. Maintaining optimal BCS is critical for the productive performance of high-yielding cows, particularly during the transition period (late gestation to early lactation) when energy demands rise sharply. During this phase, an excessive decline in BCS driven by Negative Energy Balance (NEB) has been confirmed to be closely associated with a range of adverse metabolic sequelae, including ketosis and hepatic lipidosis. Concurrently, this state of physiological stress compromises reproductive function, typically manifested as prolonged postpartum anestrus and reduced conception rates [2].

Traditional BCS methods rely on manual subjective evaluation of subcutaneous fat reserves at specific anatomical locations through visual inspection and palpation, primarily including the spine, ribs, tail head, and pelvic girdle (hooks and pins) [3]. However, this approach is not only labor-intensive, but its inherent subjectivity is also a primary source of inter-assessor variation, thereby compromising the reproducibility of the scoring data [4]. Furthermore, the assessment process necessitates direct physical contact with the animals, which may induce stress responses in cattle, subsequently exerting adverse effects on both operational efficiency and animal welfare. Collectively, these operational limitations impede the widespread application of BCS as a routine management tool within commercial production systems. Therefore, although the value of BCS in monitoring animal nutritional status and reproductive performance has been well established, the inherent drawbacks of traditional methods remain a critical factor constraining their universal adoption in practice.

To address these challenges, research attention has shifted toward developing more objective and automated BCS assessment methods. For instance, Azzaro et al. (2011) [5] estimated BCS by extracting anatomical landmarks from digital images and establishing a model using polynomial kernel Principal Component Analysis (KPCA). However, this system necessitated manual annotation of these landmarks during both the training and application phases, thereby limiting its degree of automation. Halachmi et al. (2013) [6] utilized thermal imaging technology to automatically extract body contours and fit a parabola, assessing the cows by calculating the mean absolute error between the contour and the parabola. Nevertheless, the initial concept of leveraging the thermal insulating properties of fat for scoring was not realized, as variations in hair coat thickness obscured the temperature differences attributable to the fat layer.

Advancements in computer technology and artificial intelligence have catalyzed the application of lightweight visual analysis in the agricultural sector. Methodologically, these visual analysis approaches can be broadly categorized into two modalities: two-dimensional (2D) and three-dimensional (3D).

Kojima et al. (2022) [7] developed a machine learning-based method to estimate the BCS of beef cattle. The system utilized a 3D camera to acquire three-dimensional surface data of the rump region, extracting four 3D body features as model inputs. To enhance classification performance, the study employed the Auto-WEKA tool to automatically select and optimize the algorithms; ultimately, the constructed AdaBoost.M1 model achieved an overall accuracy of 90% in the BCS classification task. Xiong et al. (2023) [8] utilized depth images to classify the BCS of mature beef cattle. This study employed a low-cost commercial Time-of-Flight (ToF) depth sensor to collect images from a dorsal view and extracted various biological features, such as body volume and dorsal area. To facilitate effective classification by machine learning models, BCS was divided into four intervals. Results indicated that among all tested classifiers, the Bagged Tree model performed best, achieving a True Positive Rate (TPR) exceeding 60% for cattle with a BCS between 4.75 and 5.5. Zhang et al. (2023) [9] proposed a generic automated method for the quantitative analysis of livestock body condition from 3D shapes. By processing depth images, this method leveraged the principle of point-to-point correspondence to precisely calculate shape differences between individuals and map them to BCS scores. A key innovation of this method lies in its cross-species applicability, as it has been successfully applied to dairy cows, beef cattle, and pigs. Experimental results demonstrated that for BCS assessment in dairy cows, the method achieved 100% accuracy when a scoring error tolerance of 0.5 was permitted.

Regarding two-dimensional (2D) imaging, Siachos et al. (2024) [10] developed a fully automated 2D BCS scoring system. This system utilized EfficientNet V2 as the backbone and incorporated ordinal regression to address the ordinal nature of BCS categories. Trained on a large-scale dataset, the system achieved high agreement with human expert assessments, attaining an accuracy of 84.6% within a 0.25-point error margin. Zheng et al. (2024) [11] proposed BCS-YOLO, a lightweight model based on an improved YOLOv8 architecture. This study optimized feature representation and reduced computational redundancy by introducing the Star-EMA module and the SSLDH detection head, while concurrently enhancing performance through channel knowledge distillation techniques. Results indicated that the model achieved a 9.4% increase in mAP with a 33% reduction in model size, effectively enabling high-precision automated scoring in resource-constrained environments. Li et al. (2025) [12] presented an automated BCS scoring system based on a modified YOLOv5. They enhanced feature extraction capabilities by incorporating a dual-path network and Convolutional Block Attention Modules (CBAM), and achieved a lightweight design by substituting partial standard modules with depthwise separable convolutions to reduce model parameters. With the parameter count reduced to 5.9 M, the model achieved an mAP of 91.8%.

The practical deployment of automated BCS is constrained by inherent challenges within both two-dimensional (2D) and three-dimensional (3D) visual modalities. 3D vision approaches face dual constraints: the high cost of specialized hardware and the heavy computational burden of processing pipelines. Although 2D imaging systems lower hardware economic barriers, their reliance on computationally intensive deep learning models impedes the scalable and efficient implementation of this technology in production environments [13].

While significant progress has been made in model compression through neural network lightweighting, exploring superior methodologies remains crucial to satisfy the stringent efficiency demands of real-world applications. Future research must focus on balancing high compression rates with robust predictive performance [14]. Consequently, deploying efficient and precise automated BCS systems within the complex environments of the beef cattle industry continues to pose a formidable challenge, necessitating deeper scientific exploration and technical validation.

To this end, this paper proposes EdgeBCS-YOLO, a lightweight beef cattle BCS framework specifically designed for edge computing deployment. By leveraging Unmanned Aerial Vehicle (UAV) aerial photography to construct a multi-view dataset and employing non-contact 2D visual analysis technology, this study achieves automated BCS through a deep restructuring of the YOLO11n architecture. This method not only helps reduce the subjective bias and high costs inherent in traditional manual scoring but also aims to improve performance under motion blur and complex background interference commonly encountered in farming environments, thereby contributing to a better balance between detection accuracy and real-time efficiency on resource-constrained edge devices. Although some BCS algorithms for livestock currently exist, research tailored to complex beef cattle farming scenarios that completes end-to-end real-time deployment verification on embedded hardware remains relatively scarce. Consequently, this study aims to reshape the model architecture by synergistically introducing position-sensitive fusion, texture-aware enhancement, and efficient detection mechanisms, thereby providing a low-latency, lightweight and potentially scalable edge-side solution for the field of precision livestock farming. The main contributions of this paper are as follows:

(1) Construction of a dedicated multi-view beef cattle BCS dataset. To address data scarcity, a multi-view dataset was established, and a dynamic blur augmentation strategy was introduced to simulate motion blur in real farm environments, thereby providing robustness-oriented training and evaluation conditions.
(2) Proposal of the core feature enhancement architecture for EdgeBCS-YOLO. The Position-Sensitive Feature Fusion (PSFF) module and Texture-Aware Star Module (TASM) were introduced to enhance fine-grained feature extraction and spatial localization under complex backgrounds.
(3) Design of an Efficient Grouped Detection Head (EGDH) for lightweight optimization. Based on grouping strategies, weighted residual fusion, and lightweight refinement, EGDH reduces computational redundancy and parameter count while maintaining high scoring precision.
(4) Introduction of a Focal and Global Knowledge Distillation (FGD)-based training strategy. A teacher–student distillation scheme was incorporated to further improve feature representation and detection accuracy without increasing inference overhead.
(5) Realization of real-time deployment on edge hardware. With TensorRT acceleration, the optimized model was successfully deployed on the NVIDIA Jetson Orin NX Super platform, supporting its real-time performance and practical feasibility.

## 2. Materials and Methods

### 2.1. Data Acquisition

In this study, a dataset for beef cattle BCS was constructed to facilitate object detection analysis. Data acquisition was conducted from 1 July 2025 to 22 August 2025. The data were sourced from three commercial farms located in Zhoukou City, Henan Province, China: Xiaoli Farm, Biaotai Farm, and Zhouwu Farm. The subjects were Simmental crossbred beef cattle aged 6 to 14 months.

**Figure 1:**
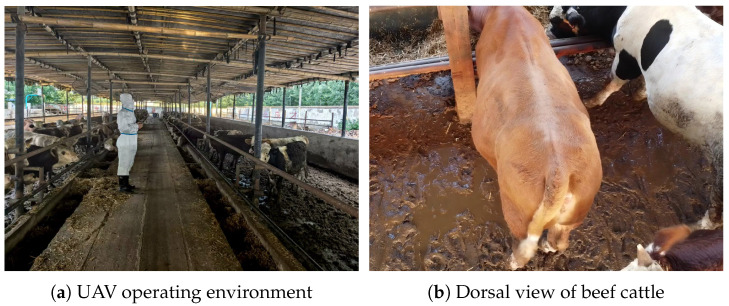
Data collection overview.

Video data were acquired using a UAV (DJI Mini 2), a method widely validated for efficient livestock monitoring [15], with the video resolution set to 1920 × 1080 pixels. To accurately capture dorsal-view images of the tailhead region, the UAV hovered at an altitude of 2.4 m above the target cattle. A gimbal tilt angle of 60° was configured to optimize the shooting perspective. The raw videos were recorded at a frame rate of 30 frames per second (FPS). A downsampling strategy—extracting one frame every three frames—was employed to construct the final dataset. This approach ensured data representativeness while enhancing processing efficiency. The on-site acquisition environment and a representative sample frame are illustrated in Figure 1.

To mitigate the impact of lighting variations on image quality, data acquisition was conducted at two fixed daily intervals (05:00 and 18:00) within the same cattle barn. In total, video data were collected from 494 beef cattle. Following frame extraction, manual screening was performed to minimize data redundancy and enhance the model’s generalization capability. The screening criteria involved discarding images with high content similarity and ensuring that eight valid images were retained for each cow from both the morning and evening sessions. Through this procedure, a final dataset comprising 7904 valid images was constructed.

### 2.2. Image Samples and Dataset

All images in the dataset were annotated by a professional veterinary team consisting of three experts in accordance with the standard 9-point BCS system. Each image was assigned a bounding box covering the tailhead region and a corresponding BCS label, thereby providing accurate annotations for model development. To ensure annotation consistency and reliability, all three annotators followed unified scoring criteria, and images with inconsistent scores were re-evaluated and resolved through discussion. Although the BCS system is defined on a 9-point scale, the present dataset includes only BCS categories 3 to 7, with cattle individuals distributed as follows: BCS 3 (17 cattle), BCS 4 (82 cattle), BCS 5 (174 cattle), BCS 6 (198 cattle), and BCS 7 (23 cattle).

To simulate image blurring induced by animal movement or insufficient camera shutter speed in real-world farming environments, data augmentation techniques were employed. Specifically, for each cattle individual, images were randomly sampled from both the morning and evening image sets, with samples drawn from both sessions and accounting for approximately one-third of the total images, and motion blur was applied along the direction of cattle movement. The size of the blur kernel was set within the range of 21 to 32, intended to simulate moderate motion blur. This procedure generated a total of 2658 blurred images.

**Figure 2:**
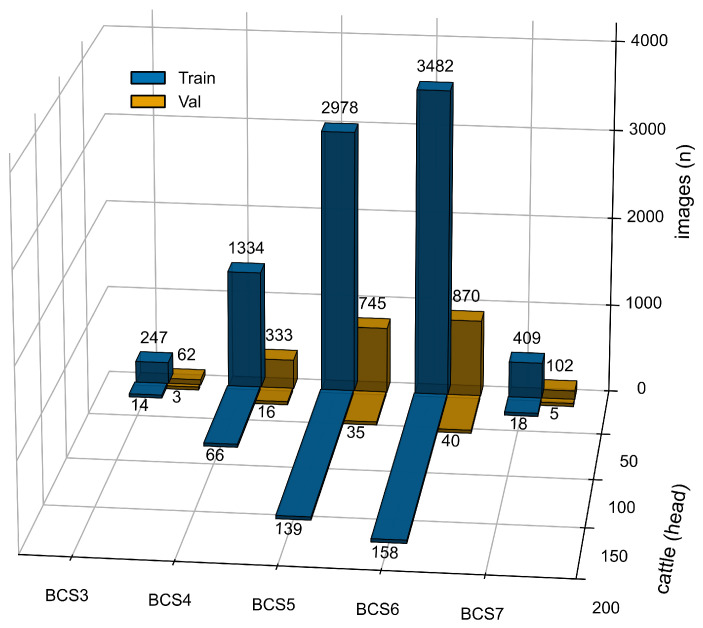
Distribution of training and validation images and cattle across different BCS categories.

Finally, the generated blurred images were integrated with the original clear images to construct a comprehensive dataset comprising 10,562 images. Within each BCS category, cattle individuals were randomly divided into the training and validation sets at a ratio of 8:2, and all images of the same individual, including both clear and blurred images, were assigned to the same subset. These two subsets were then used for model training and performance evaluation, respectively. No independent test set was established in this study due to the limited dataset size; therefore, all experimental results reported in this manuscript are based on the validation set (Figure 2).

### 2.3. Beef Cattle BCS Object Detection

#### 2.3.1. YOLO11n

The YOLO11 algorithm, released by Ultralytics, is a widely adopted model supporting various computer vision tasks. While its core architecture shares similarities with YOLOv8, it innovatively integrates the C3k2 module to enhance feature extraction. Composed of two convolutional layers, the C3k2 module represents a further optimization of the C2f module [16]. By introducing a mechanism for configurable kernel sizes, this module facilitates the extraction of features at varying levels and degrees of abstraction from the input data, thereby enhancing detection performance while simultaneously reducing computational redundancy. Furthermore, YOLO11 integrates the C2PSA module at the end of the backbone network. By incorporating a spatial attention mechanism, this module strengthens the focus on critical feature regions and effectively suppresses noise interference within complex backgrounds, further improving feature discriminability.

The network architecture of YOLO11 centers on a single-input design. The operational workflow commences with the ingestion of image data, followed by a series of data augmentation procedures, including Mosaic augmentation, random scaling, cropping, and color adjustment. These augmentation strategies enhance data diversity, thereby improving the model’s generalization capabilities [17]. Subsequently, images are resized to fixed input dimensions (640×640) and undergo pixel value normalization, typically mapping values to the [0, 1] range. This process ensures training stability and accelerates convergence. During the feature extraction phase, image data is processed using meticulously designed convolutional kernels and Batch Normalization (BN) techniques. A distinctive feature of YOLO11 is its enhanced feature extraction architecture, which emphasizes the fusion of feature information across varying scales. Through specialized structural optimization, the model efficiently processes small-scale (20×20), medium-scale (40×40), and large-scale (80×80) targets, addressing the requirements for detecting objects of varying dimensions and levels of detail. Throughout the processing pipeline, multi-scale features are effectively integrated and evaluated by core network components. Ultimately, the comprehensive detection results not only reflect YOLO11’s superior analytical capability in object detection tasks but also highlight its exceptional performance in multi-scale information fusion. Furthermore, as the Nano version within the YOLO11 series, YOLO11n maintains high detection precision while possessing an extremely low parameter count and computational complexity. This characteristic renders it highly suitable for deployment on resource-constrained edge computing devices, capable of satisfying stringent requirements for real-time performance and low latency in practical applications, thereby achieving an optimal trade-off between speed and accuracy.

#### 2.3.2. Coordinate Attention Mechanism

In object detection tasks for beef cattle BCS, accurate bounding box regression is highly dependent on the precise localization of key anatomical landmarks (e.g., the tailhead and ischial tuberosity). However, traditional Feature Pyramid Networks (FPN) typically aggregate contextual information via global pooling or large-stride convolutions during multi-scale feature fusion. While these operations effectively capture semantic information, the successive downsampling and upsampling processes inevitably induce a loss of spatial positional information. Consequently, this hinders the model’s ability to precisely localize key regions when confronted with complex backgrounds (e.g., interference from cattle pens and feces) or object occlusion. Furthermore, standard channel attention mechanisms (such as SE modules), while capable of modeling inter-channel dependencies, disregard the spatial structure of feature maps and thus fail to effectively suppress background noise interference [18].

To address the aforementioned issues, this study introduces the Coordinate Attention (CA) mechanism during the feature fusion stage [19]. Distinct from traditional global pooling, the CA mechanism embeds positional information into channel attention, thereby preserving precise spatial coordinate information while simultaneously capturing cross-channel information interactions.

Let the input feature tensor be denoted as $x\in R^{C\times H\times W}$. The CA module first employs two one-dimensional (1D) global average pooling operations to aggregate the input features along the horizontal (X-axis) and vertical (Y-axis) directions, respectively. Specifically, for the *c*-th channel, the outputs at height *h* and width *w* can be formulated as follows:

(1) $$z_{c}^{h}\left(h\right)=\frac{1}{W}\sum_{0\leq i<W}x_{c}\left(h,i\right)$$

(2) $$z_{c}^{w}\left(w\right)=\frac{1}{H}\sum_{0\leq j<H}x_{c}\left(j,w\right)$$

The aforementioned transformation decomposes global spatial information into a pair of direction-aware feature maps, which capture long-range dependencies along one spatial direction while simultaneously preserving precise positional information along the other. Subsequently, these two generated feature maps are concatenated and fed into a shared 1×1 convolutional transformation function $F_{1}$, yielding the intermediate feature map *f*:

(3) $$f=\delta (BN\left(F_{1}\left(\left[z^{h},z^{w}\right]\right)\right))$$

where [·, ·] denotes the concatenation operation along the spatial dimension, and δ represents a non-linear activation function. In our implementation, the Hardswish activation function is adopted to balance non-linear expressive capability with computational efficiency. Furthermore, BN is introduced following the convolutional layer to accelerate training convergence and enhance model stability. The resulting feature *f* is then split into two independent tensors, $f^{h}$ and $f^{w}$, which undergo two convolutional transformations, $F_{h}$ and $F_{w}$, respectively, to restore the channel dimensions. This process ultimately generates a pair of attention weight vectors, $g^{h}$ and $g^{w}$:

(4) $$g^{h}=\sigma \left(F_{h}\left(f^{h}\right)\right)$$

(5) $$g^{w}=\sigma \left(F_{w}\left(f^{w}\right)\right)$$

where σ denotes the Sigmoid activation function. Finally, the CA module performs element-wise multiplication of these two directional attention weights with the original input features, outputting the position-sensitive fused feature *y*:

(6) $$y_{c}\left(i,j\right)=x_{c}\left(i,j\right)\times g_{c}^{h}\left(i\right)\times g_{c}^{w}\left(j\right)$$

**Figure 3:**
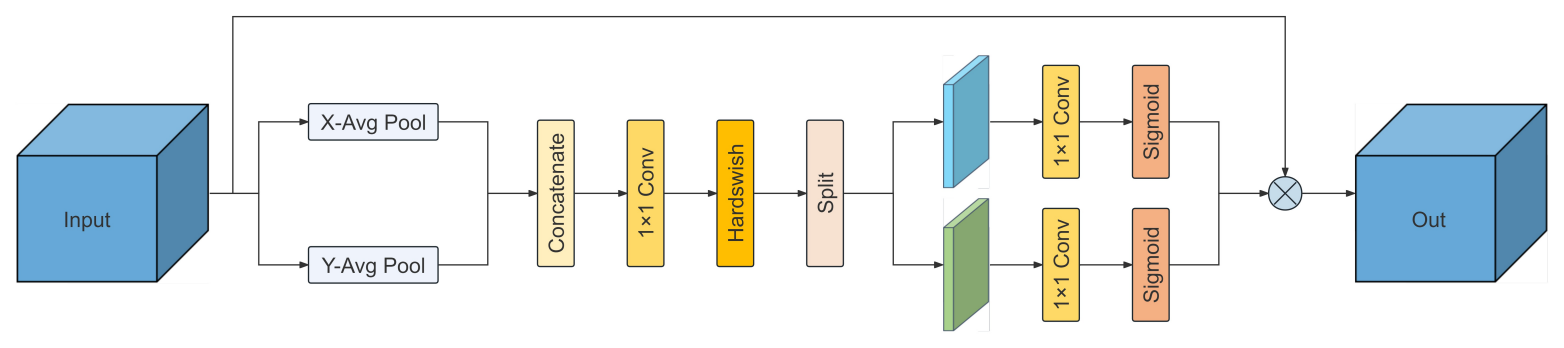
Architecture of the CA module. X-Avg Pool and Y-Avg Pool denote 1D global average pooling along the horizontal and vertical directions, respectively.

Through this mechanism, the CA module not only captures cross-channel information interactions but also effectively perceives and localizes the spatial positions of Regions of Interest (ROI). During the feature fusion process, this positional sensitivity enables the model to focus more intensively on critical anatomical parts of the beef cattle while suppressing background noise, thereby significantly improving detection robustness and localization accuracy (Figure 3).

#### 2.3.3. StarNet

**Figure 4:**
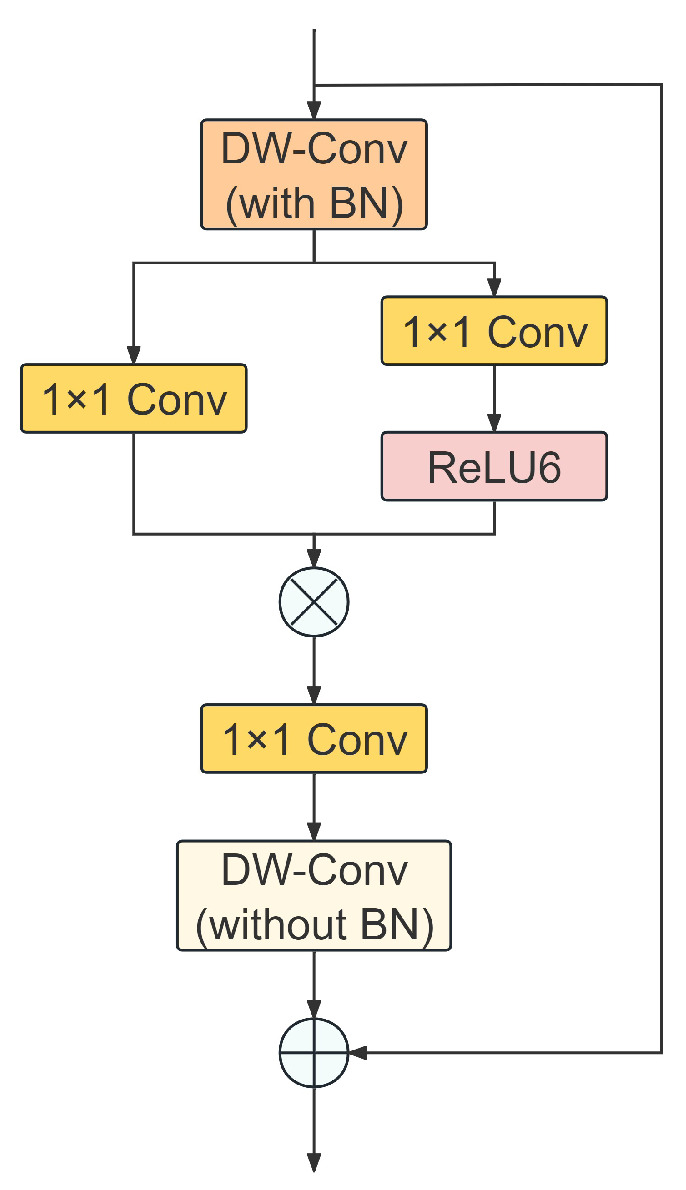
Structure of the StarNet. DW-Conv (with BN) denotes a 7×7 depth-wise convolution followed by Batch Normalization, while DW-Conv (without BN) represents a 7×7 depth-wise convolution without Batch Normalization.

In the feature extraction process for beef cattle BCS, standard Convolutional Neural Network (CNN) modules typically rely on stacking linear convolution operators with non-linear activation functions to aggregate spatial and channel information. However, this paradigm possesses inherent limitations when processing low-contrast subtle fat textures. The linear weighting mechanism of standard convolutions tends to smooth high-frequency details, hindering the model’s ability to effectively capture texture features that characterize subtle BCS variations, such as the blurred boundaries of intercostal spaces or fat coverage at the tailhead. To address this issue, this study employs StarNet [20], the structure of which is illustrated in Figure 4. By introducing the “Dual Large-Kernel Spatial Enhancement” architecture and the “Star Operation” mechanism, StarNet constructs a high-dimensional non-linear implicit feature space. This approach achieves precise perception of global anatomical structures while simultaneously enhancing fine-grained texture discriminability.

Let the input feature tensor be denoted as $X\in R^{C\times H\times W}$, where *C*, *H*, and *W* represent the number of channels, height, and width, respectively. To overcome the inherent limitations of traditional point-wise convolutions in capturing spatial context, StarNet first introduces a pre-spatial encoding phase. This phase utilizes a Large-Kernel Depthwise Convolution to preprocess input features, aiming to expand the effective Receptive Field to capture the global anatomical structure of the beef cattle. This spatial aggregation process can be formally expressed as:

(7) $$X_{pre}={\mathrm{DWConv}}_{in}^{7\times 7}\left(X\right)$$

where ${\mathrm{DWConv}}_{in}^{7\times 7}$ denotes a depthwise convolution with a kernel size of 7×7. This layer explicitly integrates BN to normalize the input feature distribution, thereby accelerating convergence for the subsequent high-order mapping.

Subsequently, StarNet performs the core Star Operation based on $X_{pre}$. To construct a high-dimensional feature space, the input features are split into two independent and parallel linear transformation branches. These two branches project the features into a latent space via Point-wise Convolution, with the transformation processes defined as:

(8) $$X_{1}=W_{1}*X_{pre}+b_{1}$$

(9) $$X_{2}=W_{2}*X_{pre}+b_{2}$$

where ∗ denotes the convolution operation, $W_{1},W_{2}\in R^{C^{\prime }\times C}$ (where $C^{\prime }$ represents the dimension after expansion) are the weight matrices, and $b_{1}$, $b_{2}$ are the bias vectors. StarNet fuses these two branches through non-linear activation and Element-wise Multiplication, mapping the features to an implicit hyperplane with dimensions increasing quadratically:

(10) $$Y_{star}=\sigma \left(X_{1}\right)⊙X_{2}$$

where ⊙ represents the Hadamard product, and σ(·) denotes the ReLU6 activation function. To theoretically elucidate how this operation enhances texture perception, we can employ Taylor expansion to approximate the aforementioned Star Operation as a polynomial form. For any single-channel output $Y_{star}^{\left(k\right)}$ in the feature space, its computation is essentially equivalent to a linear combination of second-order interaction terms of the input feature components:

(11) $$y_{star}^{\left(k\right)}\approx \sum_{(i,j)\in \Omega }w_{i,j}^{\left(k\right)}x_{i}x_{j}+O\left(x^{3}\right)$$

where $x_{i}$ and $x_{j}$ represent distinct channel components within the input feature vector, Ω denotes the set of indices for inter-channel interactions, $w_{i,j}^{\left(k\right)}$ is the weight coefficient for the second-order interaction term corresponding to the *k*-th output channel, and $O(x^{3})$ represents the high-order remainder terms in the Taylor expansion. This second-order interaction term based on $x_{i}x_{j}$ introduces an implicit high-dimensional mapping, rendering complex texture features that were originally linearly inseparable in low-dimensional space linearly separable in the high-dimensional space, thereby significantly enhancing the model’s sensitivity to subtle texture details.

Distinct from conventional designs, to eliminate potential Spatial Discontinuity introduced by point-wise convolution and further smooth texture features, StarNet introduces a post-spatial refinement stage following the Star Operation. This stage first restores channel dimensions via a linear projection layer $W_{out}$, followed immediately by a cascaded second large-kernel depthwise convolution. Notably, StarNet employs an asymmetric design strategy here: “BN before, no BN after.” Unlike the pre-encoding phase, the post-refiner ${\mathrm{DWConv}}_{out}^{7\times 7}$ utilizes pure convolution without a BN layer. This design avoids amplitude distortion to the reconstructed high-frequency texture details caused by normalization operations, ensuring that the output features preserve linear responses while fusing smoothly with the residual path:

(12) $$Y_{proj}=W_{out}*Y_{star}$$

(13) $$Y_{refined}={\mathrm{DWConv}}_{out}^{7\times 7}\left(Y_{proj}\right)$$

where $Y_{proj}$ denotes the transitional feature compressed back to the original channel dimension via the linear projection layer, and $Y_{refined}$ represents the spatially refined feature after smoothing by the second large-kernel convolution. Finally, StarNet combines the refined high-order texture features with the original input information, outputting the final feature tensor *Y* through a Residual Connection, thereby achieving dual enhancement of texture details and structural information:

(14) $$Y=Y_{refined}+X$$

In summary, by constructing a closed-loop pathway of “large-kernel preprocessing—star texture mapping—large-kernel post-refinement” combined with an asymmetric normalization strategy, StarNet effectively resolves the issue of texture feature loss encountered by standard convolutions in beef cattle BCS tasks. It significantly enhances the robustness of feature extraction for subtle subcutaneous fat variations while preserving global structural information.

### 2.4. Optimization Strategy

While generic object detection algorithms excel in conventional tasks, adapting them to edge devices for beef cattle BCS encounters formidable challenges. At the perceptual level, critical subcutaneous fat texture features are typically characterized by low contrast and susceptibility to environmental interference, limiting the capacity of traditional lightweight networks to extract fine-grained representations. At the computational level, the resource constraints of edge devices impose stringent demands on model parameter efficiency and real-time performance. To address this dilemma of balancing accuracy and efficiency, this study reconstructs a lightweight architecture tailored for edge deployment—EdgeBCS-YOLO—based on the YOLO11n framework. Through multi-dimensional deep customization of the backbone, neck, and detection head, this architecture systematically reduces computational load while significantly enhancing the model’s perceptual and discriminative capabilities regarding subtle physical cues.

#### 2.4.1. PSFF

The traditional YOLO11 neck network relies on nearest neighbor interpolation and channel concatenation to execute multi-scale feature aggregation. However, within the context of the beef cattle BCS task, this linear aggregation paradigm presents significant limitations. On one hand, the feature dimension redundancy resulting from channel concatenation triggers a surge in computational overhead, severely constraining real-time inference efficiency on edge devices. On the other hand, this mechanism struggles to effectively suppress noise interference from complex backgrounds (e.g., fences and the ground). Furthermore, during the feature resampling process, spatial positional information regarding key anatomical landmarks (such as the tailhead and ischial tuberosity) is prone to loss, thereby weakening the model’s discriminative capability for subtle physical signs.

To address the aforementioned limitations, this study reconstructs the neck network architecture and proposes the PSFF module. Given that BCS relies heavily on visual cues from specific anatomical parts, we embedded CA modules at the lateral connections of the feature pyramid. By injecting precise position-aware information into the feature stream to explicitly calibrate the spatial response distribution, this guides the network to focus attention weights on discriminative anatomical landmark regions. Simultaneously, to reduce the computational load for edge deployment and enhance feature purity, PSFF abandons high-redundancy channel concatenation in favor of a gated fusion mechanism based on element-wise multiplication and addition. This strategy utilizes high-level semantic features as “gating signals” to effectively suppress activation responses in non-target regions via non-linear modulation, achieving multi-scale information complementarity while maintaining constant channel dimensions. Furthermore, addressing the aliasing effects and checkerboard artifacts prone to be introduced by nearest neighbor interpolation, learnable Transposed Convolution is introduced to perform upsampling operations. This adaptively recovers spatial details of feature maps in a data-driven manner, generating high-resolution feature representations with sharper edges and more coherent textures. Through this explicit spatial location modeling and efficient gated fusion mechanism, the PSFF module significantly improves the model’s capture capability and robustness regarding subtle body condition features in complex farming environments, while substantially compressing computational redundancy.

#### 2.4.2. TASM

To address the challenge in beef cattle BCS tasks where subcutaneous fat texture features are faint and prone to being smoothed by traditional convolutions, this study proposes the TASM to replace the C3k2 bottleneck layer in YOLO11. The traditional C3k2 module often exhibits “low-pass filtering” characteristics when processing low-contrast images, leading to the loss of critical high-frequency details required to distinguish between adjacent scores. To this end, TASM seamlessly integrates the core architectural paradigm of StarNet into the feature extraction network. Leveraging its implicit high-dimensional mapping mechanism, TASM significantly enhances the non-linear expressive capability regarding subtle variations in fat thickness on the back and tailhead, rendering fine-grained features that were originally linearly inseparable in low-dimensional space more separable.

Simultaneously, TASM is capable of aggregating contextual information from a broader spatial range. By simulating the wide-coverage characteristic of “palpation” in manual scoring to calibrate features, it effectively suppresses interference from environmental noise, such as cattle pen occlusions, while focusing on local texture details. Furthermore, benefiting from the lightweight design of depthwise separable convolutions and redundancy-free activation functions, this module effectively controls computational load while achieving a qualitative enhancement in feature extraction capability. This architectural optimization, balancing accuracy and efficiency, enables the model to significantly bolster the non-linear expression of low-contrast subcutaneous fat textures while maintaining the original inference efficiency, thereby achieving an optimal balance between the depth of feature capture and the consumption of computational resources.

#### 2.4.3. EGDH

In single-stage object detection architectures, the detection head assumes the critical task of mapping feature maps to class probabilities and bounding box coordinates. However, the Decoupled Head structure employed by the YOLO11 baseline comprises multiple stacked standard convolutional layers, resulting in the parameter count and Floating Point Operations (FLOPs) of the head accounting for a disproportionately high percentage of the model’s total load. For edge devices with constrained computational and memory resources, this “Top-heavy” architectural design constitutes a major computational bottleneck for real-time inference.

To overcome this efficiency constraint, this study designs the EGDH. The core design philosophy of EGDH involves leveraging the sparse connectivity characteristics of Grouped Convolution (GC) to replace the dense connectivity of standard convolutions, thereby reconstructing the feature decoding pathway [21]. Specifically, EGDH introduces a lightweight Stem Module at each feature level, composed of two stacked 3×3 GC layers. To achieve an optimal balance between feature extraction independence and correlation, we dynamically fix the channel count of each convolution group at 16. Consequently, for an input feature map with *C* channels, the number of groups is dynamically set to $g=\frac{C}{16}$. From a theoretical computational perspective, assuming a kernel size of K×K and that both input and output channel counts are *C*, the FLOPs for standard convolution can be expressed as:

(15) $${\mathrm{FLOPs}}_{std}=H\times W\times K^{2}\times C^{2}$$

After introducing the grouping strategy, the computational volume is significantly reduced to:

(16) $${\mathrm{FLOPs}}_{GC}=H\times W\times K^{2}\times \frac{C^{2}}{g}=\frac{1}{g}\times {\mathrm{FLOPs}}_{std}$$

This derivation intuitively demonstrates that the grouping strategy can reduce the theoretical computational complexity to $\frac{1}{g}$ of that of standard convolutions, thereby achieving significant lightweighting.

**Figure 5:**
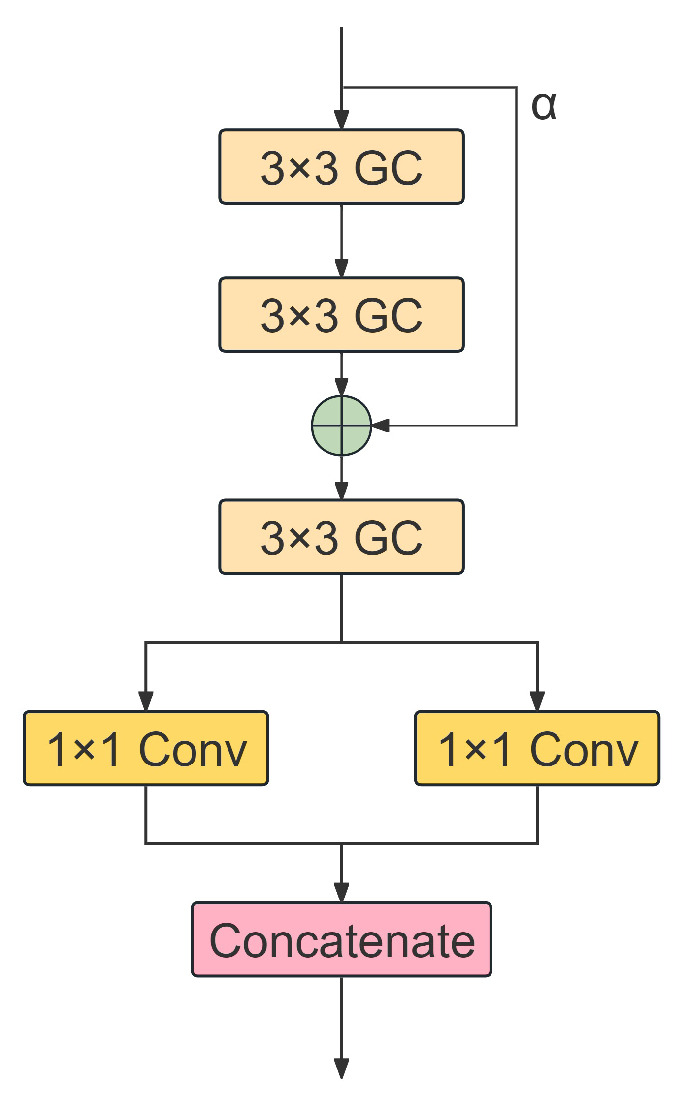
Architecture of the EGDH with weighted residual fusion and lightweight refinement.

At the architectural level, EGDH discards redundant deep stacking structures. On this basis, the input identity feature is fused with the output of the Stem Module through a learnable scaling factor α, and the fused feature is further refined by an additional 3×3 GC layer before prediction. We tightly couple BN within the grouped Stem Module and refinement branch to maintain feature distribution stability during training and enhance non-linear expressive capability. Subsequently, the refined feature maps are directly mapped to regression and classification tensors via 1×1 convolutions. This design not only avoids the distributional interference of normalization operations on final regression values but also allows for the lossless fusion of BN parameters into convolutional weights during the inference phase, thereby further reducing memory access latency during deployment. Through this reshaped convolution paradigm, EGDH effectively retains regression precision for beef cattle body condition features while substantially compressing model parameters and computational load, providing key structural support for the efficient operation of EdgeBCS-YOLO on the edge (Figure 5).

#### 2.4.4. EdgeBCS-YOLO

**Figure 6:**
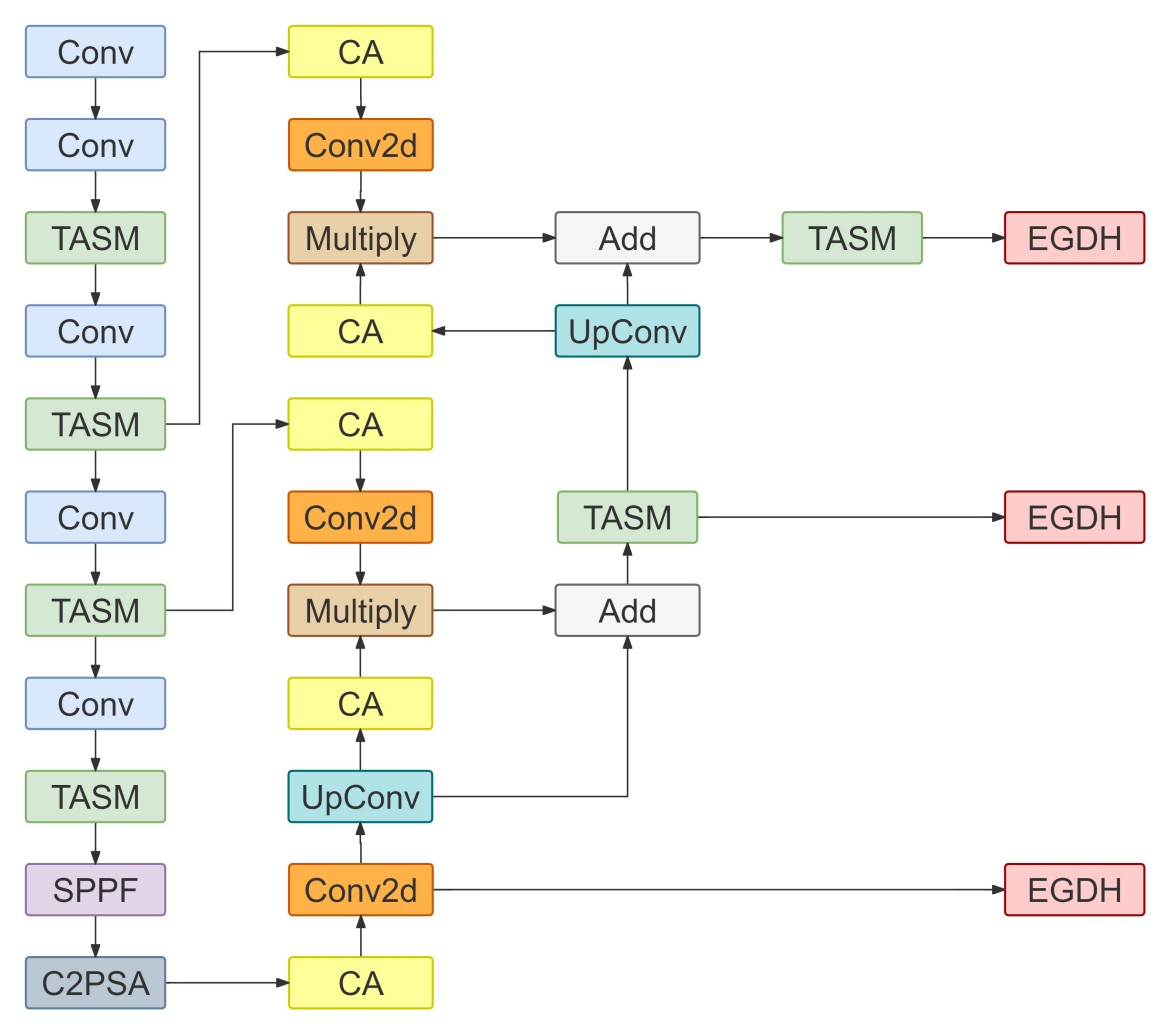
The complete architecture of EdgeBCS-YOLO. Conv2d denotes the standard 2D convolution operation, while UpConv represents the transposed convolution for learnable upsampling. Add and Multiply refer to element-wise addition and element-wise multiplication, respectively.

The complete architecture of EdgeBCS-YOLO is illustrated in Figure 6. Its core design centers on the synergistic optimization of the backbone, neck, and detection head to accommodate resource-constrained edge environments. Specifically, the TASM module replaces the original C3k2 bottleneck layers in the feature extraction network. This modification leverages high-dimensional implicit mapping to significantly enhance the model’s non-linear expressive capability regarding subtle subcutaneous fat textures. In the feature fusion stage, the model adopts the PSFF module, which substitutes high-redundancy channel concatenation with a gated fusion mechanism combined with CA. This approach preserves the precise spatial localization of key anatomical landmarks while efficiently aggregating multi-scale features. Finally, the original detection head is substituted with the lightweight EGDH. By utilizing grouped convolutions, weighted residual fusion, and lightweight feature refinement, this component effectively mitigates computational redundancy and reduces deployment overhead, thereby supporting high-frame-rate inference on devices such as the NVIDIA Jetson Orin NX Super.

#### 2.4.5. Knowledge Distillation Strategy

To further enhance the feature representation capability of the lightweight model without increasing inference cost, a Focal and Global Knowledge Distillation (FGD) strategy was introduced during training [22]. YOLO11l was used as the teacher model, while EdgeBCS-YOLO served as the student model. Distillation was applied to the neck feature maps, denoted as $F^{T}$ and $F^{S}$, respectively.

**Figure 7:**
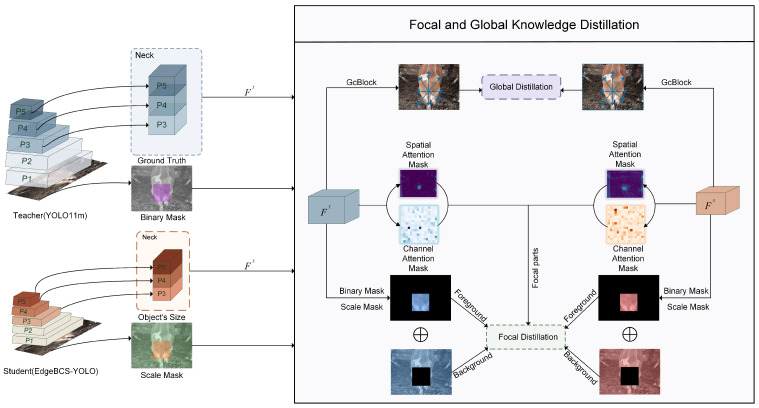
FGD-based knowledge distillation framework.

As shown in Figure 7, the distillation framework consists of focal distillation and global distillation branches. In the focal branch, the ground-truth annotations were used to generate a binary mask and a scale mask, which guided the student model to focus on key foreground anatomical regions. Spatial and channel attention masks were further introduced to emphasize informative feature responses during teacher–student transfer. In the global branch, the contextual dependencies embedded in the teacher features were transferred to the student features to improve global representation under complex farm backgrounds.

Since the distillation branch was used only during training, it introduced no additional computational cost during inference. This strategy helps enhance the discriminative capability and robustness of the lightweight model while preserving the low-latency characteristic required for edge deployment.

### 2.5. Edge Deployment Framework

To validate the deployment feasibility of EdgeBCS-YOLO in resource-constrained livestock farming scenarios, this study deployed the model on the NVIDIA Jetson Orin NX Super. Although the platform provides 117 TOPS in Super mode, its 8 GB shared memory still constrains model size and runtime memory usage for high-resolution video processing. Therefore, model lightweighting and memory optimization were necessary (Table 1).

**Figure 8:**
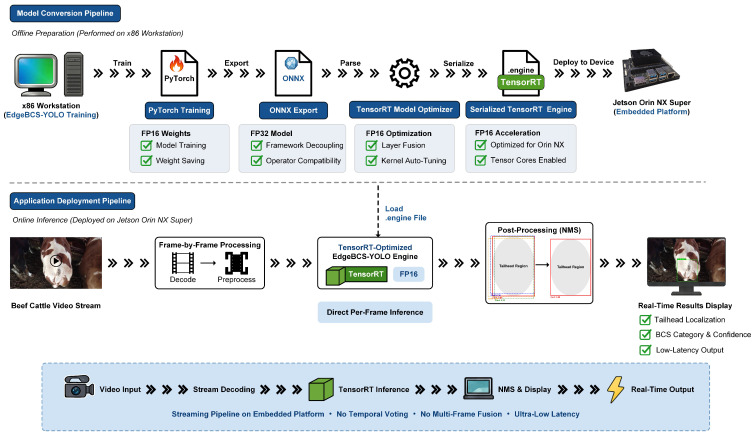
Overall deployment workflow of EdgeBCS-YOLO, including model conversion and online inference on the Jetson Orin NX Super platform.

To maximize edge-side inference throughput while ensuring operator compatibility, a model conversion and deployment pipeline was established. The model trained in PyTorch 2.8.0 was first exported to the Open Neural Network Exchange (ONNX) format with an FP32 computational graph, and the model was then parsed and reconstructed by the NVIDIA TensorRT inference engine with FP16 acceleration enabled. Through layer fusion and kernel auto-tuning, intermediate FP32 operators were remapped to FP16 kernels optimized for the Orin NX architecture, thereby fully exploiting the onboard Tensor Cores [23]. In practical deployment, beef cattle videos were processed in a streaming manner on the embedded platform. The input video was decoded frame by frame, and each frame was sequentially preprocessed and fed into the TensorRT-optimized EdgeBCS-YOLO engine for inference, which outputs the tailhead localization result, BCS category, and confidence score. After Non-Maximum Suppression (NMS) was applied to remove redundant prediction boxes, the final results were displayed in real time. This framework enabled direct per-frame online inference without temporal voting or multi-frame fusion, thus preserving the low-latency advantage required for practical edge deployment (Figure 8).

### 2.6. Experimental Settings and Training Strategies

First, Table 2 and Table 3 present the specifications of the experimental platform and the basic training parameters utilized in this study.

Key additional parameter settings are detailed as follows. To balance model convergence speed with training stability, the initial learning rate of the Stochastic Gradient Descent (SGD) optimizer was set to 0.01, with a momentum of 0.937 and a weight decay of 0.0005. To enhance robustness and generalization capabilities in complex environments, a comprehensive set of data augmentation and regularization parameters was configured. Regarding color augmentation, the coefficients hsv_h, hsv_s, and hsv_v—governing hue, saturation, and value adjustments—were set to 0.015, 0.7, and 0.4, respectively. In terms of geometric transformations, the translation and scaling parameters were fixed at 0.1 and 0.5, respectively, while horizontal flipping was enabled with a probability of 0.5 to augment viewpoint diversity. Furthermore, the Mosaic augmentation strategy was applied with a probability of 1.0 and remained active throughout the entire 100-epoch training duration to continuously enrich background contextual information. During the inference and validation phases, the Intersection over Union (IoU) threshold for Non-Maximum Suppression (NMS) was established at 0.7, effectively reducing redundant overlapping bounding boxes and ensuring detection reliability. Under these rigorous configurations, the model was trained and evaluated under consistent experimental settings.

### 2.7. Evaluation Indicators

To comprehensively evaluate the effectiveness and edge deployment potential of the proposed algorithm, this study employs a multi-dimensional evaluation framework encompassing detection accuracy, computational efficiency, and edge performance.

Regarding detection accuracy, Precision (*P*), Recall (*R*), and mAP were selected as core metrics. Precision quantifies the proportion of true positive samples among the model’s positive predictions, reflecting the model’s precision capability. Recall measures the proportion of correctly identified positive samples out of all actual positive samples, reflecting the model’s sensitivity (or recall capability). The calculation formulas for *P*, *R*, AP, and mAP are as follows:

(17) $$P=\frac{T_{P}}{T_{P}+F_{P}}\times 100\%$$

(18) $$R=\frac{T_{P}}{T_{P}+F_{N}}\times 100\%$$

(19) $$AP=\int_{0}^{1}P\left(R\right)dR$$

(20) $$mAP=\frac{1}{M}\sum_{k=1}^{M}AP\left(k\right)$$

where $T_{P}$, $F_{P}$, and $F_{N}$ represent True Positives, False Positives, and False Negatives, respectively, and *M* denotes the total number of classes. Furthermore, mAP@50 (IoU = 0.5) is utilized to assess basic localization accuracy, whereas mAP@50:95 (IoU = 0.5:0.05:0.95) reflects the model’s comprehensive performance in high-precision bounding box regression tasks [24].

In terms of model efficiency, GFLOPs is employed to measure computational complexity, while Model Size is used to evaluate storage requirements.

To assess the potential for edge deployment, Model Size is utilized to evaluate storage overhead. To quantify real-time inference performance, Average Inference Latency ($t_{latency}$) and Theoretical FPS are introduced. Specifically, Theoretical FPS characterizes the theoretical upper bound of computing power, calculated as follows:

(21) $${\mathrm{FPS}}_{theoretical}=\frac{1000}{t_{latency}}$$

Furthermore, System FPS is employed to evaluate the model’s real-time response capability under end-to-end practical operating conditions.

## 3. Results

### 3.1. Ablation Experiments

To systematically evaluate the independent contributions and synergistic effects of the proposed modules on the overall network performance, a comprehensive ablation study was designed and conducted using YOLO11n as the baseline. Adopting a step-wise integration strategy, the PSFF, TASM, and EGDH modules were sequentially incorporated, followed by an additional distillation stage based on FGD. To comprehensively assess the efficiency-accuracy trade-off, a multi-dimensional evaluation framework was established, encompassing GFLOPs, Model Size, Precision, Recall, mAP@50, and mAP@50:95. The detailed quantitative experimental results are presented in Table 4.

The baseline YOLO11n model yielded 6.3 GFLOPs, a Model Size of 5.2 MB, Precision of 87.9%, Recall of 79.1%, mAP@50 of 84.8%, and mAP@50:95 of 56.2%. Upon this foundation, the introduction of the PSFF module first optimized the feature fusion process, significantly reducing model redundancy by compressing the Model Size to 3.7 MB and lowering GFLOPs to 5.6. Although Precision improved to 88.8% at this stage, and mAP@50 and mAP@50:95 rose to 85.3% and 56.6% respectively, it is noteworthy that Recall experienced a slight decline from 79.1% to 77.4%. This data indicates that while PSFF improved detection accuracy by suppressing background noise, its relatively conservative feature screening strategy filtered out some ambiguous features to a certain extent, resulting in fluctuations in the capability to capture hard samples.

Subsequently, the integration of the TASM module effectively resolved this Recall bottleneck. Maintaining the computational load at 5.6 GFLOPs, this module further reduced the Model Size marginally to 3.6 MB. More critically, TASM successfully reversed the downward trend in Recall, significantly restoring it to 78.5% while simultaneously pushing Precision to 89.3%. Driven by this, mAP@50 and mAP@50:95 also grew to 85.5% and 56.7%, respectively. This comprehensive performance recovery confirms that TASM’s high-order feature mapping mechanism can significantly enhance the extraction of fine-grained somatic features of beef cattle without adding extra computational overhead, thereby effectively recovering missed targets.

After replacing the original detection head with EGDH, the model achieved a strong balance between performance and efficiency. This improvement reduced GFLOPs to 4.7 and Model Size to 3.3 MB. Meanwhile, Precision, Recall, mAP@50, and mAP@50:95 reached 89.7%, 79.8%, 86.7%, and 57.1%, respectively, confirming that EGDH effectively improves detection capability while maintaining a lightweight architecture.

On this basis, the distilled variant further improved the overall performance without introducing additional inference overhead. Specifically, EdgeBCS-YOLO (YOLO11n-PSFF-TASM-EGDH (Distilled)) maintained the same GFLOPs and Model Size as the non-distilled counterpart, while increasing Precision, Recall, mAP@50, and mAP@50:95 to 90.8%, 82.7%, 88.9%, and 59.8%, respectively. These results demonstrate that the introduced distillation strategy further enhances the student model’s feature representation and detection accuracy under the same deployment cost.

**Figure 9:**
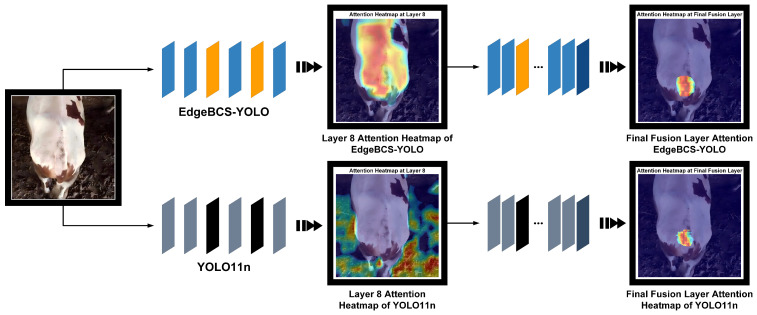
Attention heatmap comparison of YOLO11n and EdgeBCS-YOLO at key layers.

To intuitively validate the effectiveness of the proposed modules from a qualitative perspective, Figure 9 presents a comparison of attention heatmaps between the baseline and improved models at two key depths: the Deep Backbone Layer (representing TASM output features) and the Final Feature Fusion Layer (representing EGDH input features).

At the deep backbone layer (Layer 8), the baseline model’s attention distribution exhibits significant divergence, with numerous high-response regions erroneously scattered across background noise such as the pen floor. In contrast, the improved model integrated with TASM demonstrates superior anti-interference capability, where its activation regions are highly concentrated and precisely aligned with the dorsal spine line of the cattle. This phenomenon visually confirms that TASM effectively suppresses environmental background noise by constructing high-order texture mappings and forces the backbone network to focus on extracting robust somatic semantic features.

At the final feature fusion layer, the model integrated with the PSFF module showcases a significant performance leap, generating attention heatmaps with concentrated intensity and distinct contours. Conversely, the baseline model’s response is often limited to vague and discrete local features. By effectively aggregating multi-scale contextual information and performing spatial calibration via the PSFF module, the improved model achieves enhanced semantic localization capabilities, enabling it to precisely lock onto and cover the anatomical boundaries of the key tailhead region. This high-quality qualitative visualization provides an intuitive explanation for the improvements in mAP@50 and mAP@50:95 observed in the quantitative experiments, confirming that this feature layer possesses sufficient high-order semantic discriminability to lay a solid foundation for the detection head to output high-precision bounding boxes.

### 3.2. Comparative Analysis

To comprehensively evaluate the performance of EdgeBCS-YOLO, comparative experiments were conducted against existing mainstream object detection algorithms. The comparative baselines encompass representative models across varying computational scales, including the classic single-stage detector SSD [25], the Transformer-based RT-DETR-L [26], and a series of lightweight YOLO models tailored for mobile deployment (YOLOv5n, YOLOv6n [27], YOLOv7-tiny [28], YOLOv8n, YOLOv9-tiny [29], YOLOv10n [30], YOLOv12n [31], and YOLOv13n [32]). All models were trained and evaluated under identical hardware configurations and dataset partitioning strategies. The same dataset, including the motion-blurred images generated during dataset construction, was used for all compared methods. No additional illumination preprocessing was applied. Detailed comparative experimental results are presented in Table 5.

As illustrated in the table, EdgeBCS-YOLO achieves an optimal trade-off between detection accuracy and computational efficiency. Regarding computational complexity, EdgeBCS-YOLO demonstrates significant lightweight advantages. Compared with the parameter-heavy RT-DETR-L (103.5 GFLOPs) and SSD (30.63 GFLOPs), the GFLOPs of the proposed model are reduced by 95.5% and 84.7%, respectively, enabling it to adapt to resource-constrained edge computing environments. Even within the cohort of lightweight models, the resource consumption of EdgeBCS-YOLO is significantly lower than that of the latest YOLOv12n and YOLOv13n. Specifically, compared to the widely adopted YOLOv8n, the proposed model reduces the computational load by 42.0% (from 8.1 to 4.7 GFLOPs) while simultaneously compressing the model weight size by 44.1% (from 5.9 to 3.3 MB), thereby drastically lowering the barrier for deployment.

In terms of detection performance, despite the substantial compression in parameter count, EdgeBCS-YOLO maintains competitive accuracy. Its mAP@50 reaches 88.9% and mAP@50:95 achieves 59.8%; both key metrics outperform all comparative models. Notably, the model achieves the highest Precision (90.8%), and its Recall (82.7%) also ranks first among all compared models. This indicates that EdgeBCS-YOLO does not trade high precision for sacrificed recall; rather, it significantly enhances prediction confidence and accuracy while maintaining the capability to capture hard samples. In summary, EdgeBCS-YOLO provides a competitive balance between detection performance and computational efficiency for beef cattle BCS.

### 3.3. Preliminary Robustness Evaluation

#### 3.3.1. Impact of Dynamic Blurring

**Figure 10:**
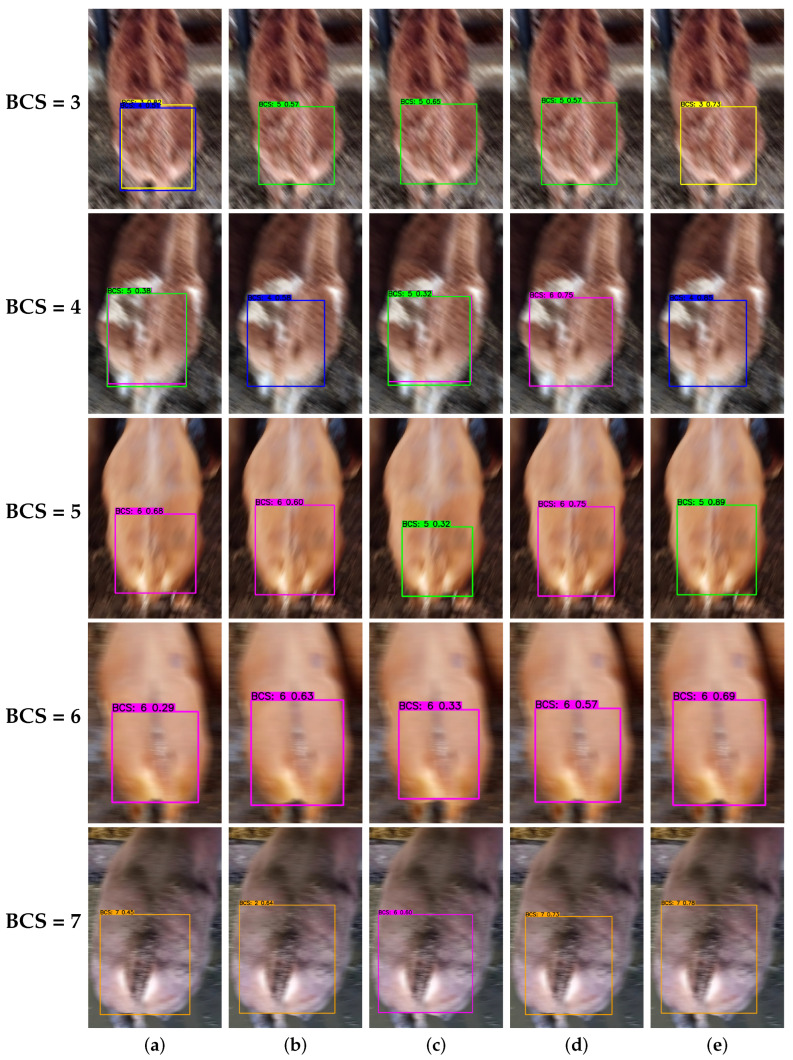
Performance comparison under 35-pixel motion blur. Rows indicate BCS classes 3–7. (**a**) SSD, (**b**) RT-DETR-L, (**c**) YOLOv8n, (**d**) YOLO11n, (**e**) EdgeBCS-YOLO (Ours).

In practical ranching operations, motion-induced image blurring often obscures critical anatomical features (e.g., the tailhead and spine), severely constraining detection performance [33]. To provide a preliminary evaluation of robustness under degraded image quality, this study applied a 35-pixel motion blur kernel to simulate a challenging blurred condition. Experiments revealed (Figure 10) that SSD and YOLOv8n, lacking fine-grained feature recovery mechanisms, exhibited conflicting redundant detections and misclassifications. While RT-DETR-L maintained localization stability due to its Transformer architecture, the loss of high-frequency textures similarly resulted in misclassifications. YOLO11n also struggled to discern weak high-frequency signals such as intercostal spaces, leading to confusion between adjacent scoring grades.

In contrast, EdgeBCS-YOLO showed more reliable detection performance under the simulated motion-blur setting, achieving accurate detections with high confidence across all scoring categories. This advantage is attributed to the synergistic optimization of the model architecture: the TASM module leverages implicit high-dimensional mapping to effectively enhance latent texture feature representation under blur degradation, compensating for the loss of high-frequency information; meanwhile, the PSFF module utilizes the CA mechanism to ensure precise localization of anatomical landmarks even when spatial details are compromised. These results suggest that the proposed model may alleviate feature-smoothing effects in blurred images and provide improved resilience to motion-related degradation. However, because the present analysis was based on simulated blur rather than broader field validation, these findings should be interpreted as preliminary evidence rather than a comprehensive assessment of robustness in real-world dynamic environments.

#### 3.3.2. Impact of Lighting Conditions

**Figure 11:**
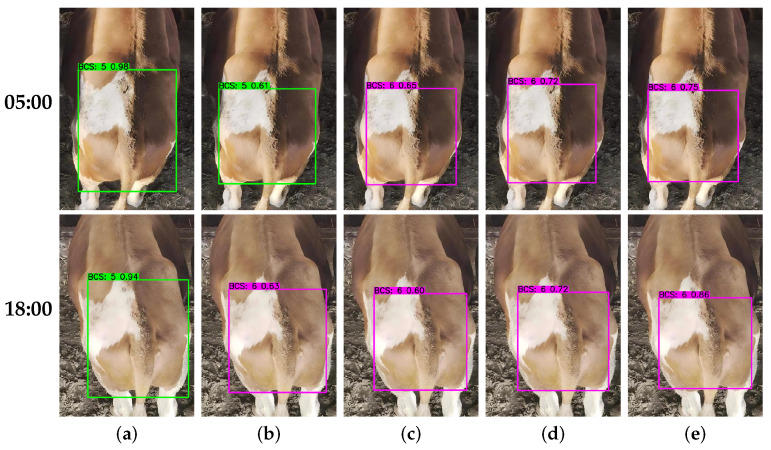
Visual comparison of detection results under different lighting conditions (accurate BCS: 6). The rows correspond to 05:00 (**top**) and 18:00 (**bottom**) environments. (**a**) SSD, (**b**) RT-DETR-L, (**c**) YOLOv8n, (**d**) YOLO11n, (**e**) EdgeBCS-YOLO (Ours).

Dynamic illumination variation significantly alters the shadow distribution of subcutaneous fat and skeletal features on cattle, constituting an important factor that may affect the reliability of automated scoring under variable field lighting conditions [34]. To provide an illustrative qualitative comparison rather than a comprehensive robustness benchmark, this study selected a single subject (BCS = 6) for comparative testing under varying lighting conditions in the morning and evening (Figure 11).

In contrast, YOLOv8n, YOLO11n, and EdgeBCS-YOLO all showed adaptability to the specific lighting cases examined here, successfully overcoming visual interference to output correct classification results. However, EdgeBCS-YOLO held a distinct advantage regarding prediction confidence score. Whether under high-contrast morning conditions or low-illumination evening settings, the proposed model maintained the highest confidence levels within the group. These observations suggest that, with the non-linear enhancement of local texture features by TASM and the precise localization of key anatomical regions by PSFF, EdgeBCS-YOLO may better reduce the influence of lighting-related visual interference in the representative cases considered here. However, because this comparison was based on a single animal and two illustrative lighting conditions, it should be regarded as preliminary qualitative evidence rather than comprehensive validation of illumination robustness in all-day operational scenarios.

### 3.4. Real-World Performance on Edge Devices

To evaluate the practical deployment efficacy of the proposed algorithm within resource-constrained livestock farming scenarios, this study established an edge inference environment based on the NVIDIA Jetson Orin NX Super embedded computing platform and conducted end-to-end performance tests. Detailed quantitative experimental results are summarized in Table 6.

Regarding storage efficiency, EdgeBCS-YOLO demonstrates significant lightweight advantages. As shown in Table 6, benefiting from the overall lightweight design of the proposed framework, the Model Size was successfully compressed to 3.95 MB. Compared to the baseline model YOLO11n (4.36 MB), this model achieves a volume reduction of 9.4%. This result indicates that the proposed framework maintains a clear advantage in static storage occupancy for edge deployment. It should be emphasized that the size of the TensorRT engine is influenced not only by the model weights, but also by the serialized network topology, tensor scheduling information, and optimization-related execution descriptors generated during compilation. As a result, although the proposed model contains fewer effective parameters, the introduction of customized structural components leads to a slight increase in non-parameter storage overhead in the final engine. This explains why the compiled deployment file is marginally larger than the original weights reported in Table 4, while still remaining smaller than the baseline after deployment. This optimization not only substantially reduces static storage occupancy on edge devices but also effectively alleviates bandwidth pressure during network transmission, providing critical convenience for remote model updates via wireless networks in remote ranch environments.

In terms of inference speed and real-time performance, although the introduction of complex attention mechanisms and feature fusion modules caused the Average Inference Latency of EdgeBCS-YOLO to increase marginally from the baseline’s 12.40 ms to 13.26 ms, this latency remains far below the threshold of human-perceptible lag. From the perspective of throughput metrics, the model’s Theoretical FPS reaches as high as 75.41 FPS, indicating that the hardware computing power retains an ample performance margin. In actual deployment, constrained by the physical bottlenecks of video capture frame rates and display refresh rates, the System FPS of EdgeBCS-YOLO and YOLO11n stabilized at 33.35 FPS and 33.53 FPS, respectively. This result confirms that EdgeBCS-YOLO is capable of achieving stable operation on the edge without sacrificing real-time performance (meeting the hard real-time requirement of 30 FPS [35]).

**Figure 12:**
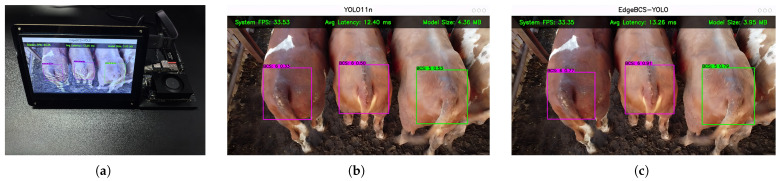
Real-world deployment and inference visualization. (**a**) Physical experimental setup with NVIDIA Jetson Orin NX Super. (**b**) Detection interface of the baseline YOLO11n. (**c**) Detection interface of the proposed EdgeBCS-YOLO. The top overlay displays System FPS, Average Inference Latency, and Model Size.

Qualitative visualization results (Figure 12) provide preliminary support for the model’s robustness. When running on the edge, EdgeBCS-YOLO is capable of locking onto moving beef cattle targets in real-time with high confidence. Furthermore, both the fitting precision of the detection boxes and the visual clarity of the labels are superior to those of the baseline model. In summary, EdgeBCS-YOLO successfully achieves a favorable balance between high precision, low storage occupancy, and real-time response on the NVIDIA Jetson Orin NX Super platform, suggesting preliminary feasibility for practical deployment on edge devices.

### 3.5. Stability Evaluation

To further assess the performance stability of the proposed model, repeated-run experiments were conducted on the baseline YOLO11n and the final EdgeBCS-YOLO. The dataset split and training settings were kept consistent with those described in the previous experiments, and each model was evaluated over five runs. The results are reported as mean ± standard deviation, and the corresponding statistical results are summarized in Table 7.

As shown in the table, both models exhibited limited variation across repeated runs, while EdgeBCS-YOLO maintained relatively small standard deviations for all evaluation metrics. This observation indicates that the proposed model maintains relatively stable detection performance under repeated experiments.

Overall, the repeated-run evaluation further supports the stability of EdgeBCS-YOLO for beef cattle BCS detection.

## 4. Discussion

### 4.1. Mechanism Analysis

The performance of EdgeBCS-YOLO results from the joint effects of localization enhancement, texture representation, efficient prediction, and distillation-guided optimization. Automated BCS assessment depends not only on accurate localization of anatomically relevant regions, but also on effective representation of subtle body-surface features [10,11,12]. From the perspective of previous PLF and automated BCS studies, this interpretation is also consistent with the broader transition from manual visual scoring toward non-contact image-based assessment. Earlier 3D- or depth-based studies showed that body-surface geometry can provide informative cues for BCS estimation, but such approaches often rely on dedicated imaging devices and relatively complex acquisition and processing pipelines [7,8,9]. By contrast, recent 2D vision-based methods can be implemented with simpler camera setups and may offer greater convenience for on-farm deployment, although their performance remains closely linked to reliable identification of anatomically informative regions and subtle visual differences associated with body condition [10,11,12].

Within this framework, PSFF improves the localization of key regions such as the tailhead and surrounding bony landmarks, allowing the detector to focus more consistently on biologically meaningful areas rather than background interference. This is biologically relevant because expert BCS assessment also relies on visual attention to specific anatomical regions where fat reserves are most evidently expressed. This interpretation is also compatible with practical BCS assessment, in which visual inspection is used to identify key anatomical regions, whereas palpation and close observation provide supplementary cues about local fat reserves. On this basis, TASM further strengthens sensitivity to subtle texture variations associated with fat deposition, including low-contrast contour and surface-texture changes, which may support more reliable discrimination of adjacent or moderate body condition differences under motion blur and complex farm backgrounds. Therefore, the improvements in localization and texture sensitivity are not only technical, but also relevant to the biological basis of BCS.

At the prediction stage, EGDH improves deployment efficiency through grouped convolutions, weighted residual fusion, and lightweight refinement, thereby reducing redundant computation while preserving regression capability on edge devices. The FGD-based strategy complements this design during training by transferring both foreground-focused and global contextual information from the teacher model to the lightweight student model. In combination, these components contribute to the observed gains in precision, recall, and mAP, indicating that the performance of EdgeBCS-YOLO arises from the coordinated effects of PSFF, TASM, EGDH, and FGD-based optimization. Compared with previous automated BCS studies that mainly emphasized sensing modality or predictive accuracy [7,8,9,10,11,12], the present work places greater emphasis on the balance among feature discrimination, lightweight design, and edge-side deployment.

### 4.2. Potential Value of Edge Computing in PLF

Beyond the accuracy improvements observed in the offline evaluation, the deployment results also indicate that EdgeBCS-YOLO may be of practical value in PLF, where real-time response, limited computing resources, and unstable network conditions are common constraints. Cloud–edge–device collaborative architectures offer clear advantages in such scenarios by reducing latency, alleviating bandwidth dependence, and improving resilience under weak rural connectivity [36]. In this setting, local inference allows image data to be processed near the source, while only compact structured results need to be transmitted when required. Such a deployment pattern is particularly relevant for routine livestock monitoring, because it enables repeated on-farm assessment without relying on continuous transmission of raw video streams to remote servers.

These deployment characteristics are also meaningful from a management perspective. Real-time and non-contact BCS monitoring may support more timely feeding adjustment, grouping, and routine condition assessment, especially when changes in fat reserves need to be tracked more consistently at the herd level. Because BCS changes are often reflected in localized anatomical and surface-texture differences, improved localization and texture sensitivity may also make such condition changes easier to detect in routine farm monitoring. At the same time, local processing can reduce the large-scale transmission of raw farm data, which further supports the potential applicability of EdgeBCS-YOLO in on-farm monitoring scenarios.

### 4.3. Limitations and Future Work

Despite the encouraging results of EdgeBCS-YOLO in real-time edge-side inference, several limitations should be acknowledged. The current dataset mainly covers BCS categories 3–7, whereas extreme categories 1–2 and 8–9 are absent. Although this distribution reflects the dominant population in commercial farms, it limits validation under severe underconditioning or excessive fat accumulation. This limitation is also related to a broader issue in PLF computer vision, where publicly available datasets remain limited in diversity and coverage of challenging real-world scenarios [37]. Therefore, the applicability of the model to extreme body condition states still requires further verification.

Another limitation concerns the current evaluation setting. The training and validation sets were partitioned at the animal level with broadly consistent category distributions, which helps reduce evaluation bias. However, substantial distribution differences could make the reported performance less representative. In addition, no independent test set was established because of the limited dataset size, and all reported results are therefore based on the validation set. Moreover, the current study reports evaluation metrics obtained under a fixed experimental setting, but the statistical validation remains limited. As a result, although repeated-run evaluation was further conducted, more comprehensive statistical validation would still be valuable. The current robustness evidence should also be interpreted with caution, since the motion-blur analysis was simulation-based and the illumination analysis was limited to an illustrative setting. Therefore, these results provide only preliminary support for robustness. Future work will require larger-scale data collection, broader class-distribution evaluation, more comprehensive statistical validation, more comprehensive robustness assessment, and independent test-set validation.

A further limitation is that the current deployment mode still focuses mainly on single-point perception and immediate output. Long-term integration of structured monitoring results has not yet been established, which limits its present value for longitudinal herd analysis and strategic feeding management. A more complete PLF workflow will likely depend on cloud–edge collaboration to connect real-time local inference with higher-level historical analysis and decision support [38]. Accordingly, future work will focus on expanding the dataset to cover the full BCS range and more diverse farm conditions, strengthening validation under broader field settings, and developing a cloud–edge collaborative management platform for long-term data analysis and visualization.

## 5. Conclusions

To address the subjectivity of manual assessment in PLF and the deployment difficulty of computationally intensive automated methods, this study proposes EdgeBCS-YOLO, a lightweight object detection model for real-time beef cattle BCS on edge devices. Built upon the YOLO11n framework, the proposed model improves the balance between detection accuracy and inference efficiency by jointly optimizing feature extraction, feature fusion, prediction efficiency, and distillation-guided training. Specifically, PSFF improves background suppression and key-region localization, TASM enhances the representation of low-contrast fat-related texture cues, EGDH reduces parameter redundancy and computational overhead, and the FGD-based strategy further improves the student model without increasing inference cost.

Experimental evaluations on the beef cattle dataset showed that EdgeBCS-YOLO achieved a precision of 90.8%, a recall of 82.7%, an mAP@50 of 88.9%, and an mAP@50:95 of 59.8%, indicating competitive performance among the compared lightweight detection models. Deployment on the NVIDIA Jetson Orin NX Super provided preliminary support for its feasibility for real-time edge-side BCS inference. With a model size of 3.95 MB, an average inference latency of 13.26 ms, and a system FPS of 33.35, the model achieved real-time operation under the current deployment setting.

Overall, EdgeBCS-YOLO provides a promising non-invasive solution for automated beef cattle monitoring for automated beef cattle monitoring, and the results support the feasibility of deploying lightweight visual models on edge computing devices without relying on high-bandwidth cloud infrastructure. Nevertheless, the current conclusions should be interpreted in light of the dataset, robustness, and evaluation limitations. The available data do not cover extreme BCS categories, the current robustness evidence remains preliminary, and all reported experimental results are based on the validation set because no independent test set was established. Future work will therefore focus on expanding the dataset to include a wider range of BCS categories and environmental variability, conducting more comprehensive robustness validation under broader field conditions, performing more rigorous independent test-set evaluation, and developing a cloud-edge collaborative management platform that integrates real-time edge inference with long-term physiological data analysis for precision breeding and nutritional management.

## Figures and Tables

**Table 1:** Specifications of the edge computing platform.

Parameters	Configuration
Device Model	NVIDIA Jetson Orin NX Super
GPU Architecture	1024-core NVIDIA Ampere GPU with 32 Tensor Cores
CPU	6-core Arm Cortex-A78AE v8.2 64-bit CPU
AI Performance	117 TOPS
Memory	8 GB 128-bit LPDDR5

**Table 2:** Specifications of the experimental platform.

Parameters	Configuration
CPU	Intel Core i5-14600KF
GPU	NVIDIA GeForce RTX 5070 Ti
Compiler	Python 3.9.21
Network construction method	PyTorch 2.8.0
Operational platform	Windows 10

**Table 3:** Key training parameters.

Parameters	Setup
Epochs	100
Batch size	16
Workers	4
Input image size	640
Optimizer	SGD

**Table 4:** Ablation and distillation results of YOLO11n-based variants.

Model	GFLOPs	Size (MB)	Precision/%	Recall/%	mAP@50/%	mAP@50:95/%
YOLO11n (Baseline)	6.3	5.2	87.9	79.1	84.8	56.2
YOLO11n-PSFF	5.6	3.7	88.8	77.4	85.3	56.6
YOLO11n-PSFF-TASM	5.6	3.6	89.3	78.5	85.5	56.7
YOLO11n-PSFF-TASM-EGDH	4.7	3.3	89.7	79.8	86.7	57.1
YOLO11n-PSFF-TASM-EGDH (Distilled)	4.7	3.3	90.8	82.7	88.9	59.8
YOLO11l (Teacher model)	86.6	48.8	92.9	86.3	91.6	63.2

**Table 5:** Comparison of performance and complexity among different detection models.

Model	GFLOPs	Size (MB)	Precision/%	Recall/%	mAP@50/%	mAP@50:95/%
SSD	30.63	93.6	80.9	59.2	80.9	50.0
RT-DETR-L	103.5	63.1	87.1	75.5	76.1	49.9
YOLOv5n	7.1	5.0	88.4	78.2	84.9	56.3
YOLOv6n	11.8	8.3	86.4	76.2	81.9	54.9
YOLOv7-tiny	13.2	11.7	81.9	74.9	80.5	50.4
YOLOv8n	8.1	5.9	84.1	80.2	84.9	56.4
YOLOv9-tiny	7.6	4.4	86.6	79.3	84.7	55.9
YOLOv10n	6.5	5.5	87.1	74.8	83.5	55.1
YOLOv12n	6.3	5.2	86.3	75.7	84.1	56.1
YOLOv13n	6.2	5.1	88.1	74.7	83.4	55.8
EdgeBCS-YOLO	4.7	3.3	90.8	82.7	88.9	59.8

**Table 6:** Performance comparison on the edge computing platform.

Model	Size (MB)	Average Inference Latency (ms)	Theoretical FPS	System FPS
YOLO11n	4.36	12.40	80.65	33.53
EdgeBCS-YOLO	3.95	13.26	75.41	33.35

**Table 7:** Repeated-run stability evaluation of YOLO11n and EdgeBCS-YOLO.

Model	Precision/%	Recall/%	mAP@50/%	mAP@50:95/%
YOLO11n	86.8 ± 0.7	78.9 ± 0.6	84.6 ± 0.3	56.2 ± 0.1
EdgeBCS-YOLO	90.7 ± 0.2	82.7 ± 0.1	88.8 ± 0.2	59.8 ± 0.1

## Data Availability

The data presented in this study are not publicly available due to privacy and ownership restrictions but may be available from the corresponding authors upon reasonable request.
